# Iron and callose homeostatic regulation in rice roots under low phosphorus

**DOI:** 10.1186/s12870-018-1486-z

**Published:** 2018-12-04

**Authors:** Yan Ding, Zegang Wang, Menglian Ren, Ping Zhang, Zhongnan Li, Sheng Chen, Cailin Ge, Yulong Wang

**Affiliations:** 1 Jiangsu Key Laboratory of Crop Genetics and Physiology/ Jiangsu Key Laboratory of Crop Cultivation and Physiology, Jiangsu Co-Innovation Center for Modern Production Technology of Grain Crops, Agricultural College of Yangzhou University, Yangzhou University, 88 Daxue South Road, Yangzhou, 225009 People’s Republic of China; 2 College of Bioscience and Biotechnology, Yangzhou University, 88 Daxue South Road, Yangzhou, 225009 People’s Republic of China; 3 College of Materials and chemical engineering, Bengbu University, 1866 Caoshan Road, Bengbu, 233000 People’s Republic of China

**Keywords:** Rice (*Oryza sativa*), Low phosphorus, Iron homeostasis, Root morphology

## Abstract

**Background:**

Phosphorus (Pi) deficiency induces root morphological remodeling in plants. The primary root length of rice increased under Pi deficiency stress; however, the underlying mechanism is not well understood. In this study, transcriptome analysis (RNA-seq) and Real-time quantitative PCR (qRT-PCR) techniques were combined with the determination of physiological and biochemical indexes to research the regulation mechanisms of iron (Fe) accumulation and callose deposition in rice roots, to illuminate the relationship between Fe accumulation and primary root growth under Pi deficient conditions.

**Results:**

Induced expression of *LPR1* genes was observed under low Pi, which also caused Fe accumulation, resulting in iron plaque formation on the root surface in rice; however, in contrast to *Arabidopsis*, low Pi promoted primary root lengthening in rice. This might be due to Fe accumulation and callose deposition being still appropriately regulated under low Pi. The down-regulated expression of Fe-uptake-related key genes (including *IRT*, *NAS*, *NAAT*, *YSLs*, *OsNRAMP1*, *ZIPs*, *ARF*, and *Rabs*) inhibited iron uptake pathways I, II, and III in rice roots under low Pi conditions. In contrast, due to the up-regulated expression of the *VITs* gene, Fe was increasingly stored in both root vacuoles and cell walls. Furthermore, due to induced expression and increased activity of β-1-3 glucanase, callose deposition was more controlled in low Pi treated rice roots. In addition, low Pi and low Fe treatment still caused primary root lengthening.

**Conclusions:**

The obtained results indicate that Low phosphorus induces iron and callose homeostatic regulation in rice roots. Because of the Fe homeostatic regulation, Fe plays a small role in rice root morphological remodeling under low Pi.

## Background

Plant root morphology is regulated by numerous factors, such as water and nutrient availability. Phosphorus (Pi) and iron (Fe) have been reported to influence the plant root length. In *Arabidopsis,* it has been proposed that Pi deficiency inhibits the root apical meristem (RAM) activity due to increased Fe bioavailability and its associated cellular toxicity [1].

The remodeling mechanism has been reported for *Arabidopsis* on root morphology in low Pi. Multicopper oxidase, Low Phosphate Root 1 (LPR1) is necessary for root growth inhibition caused by Pi limitation in *Arabidopsis*. A common pathway combining with LPR2 and PHOSPHATE DEFICIENCY RESPONSE 2 (PDR2) adjusts root meristem activity and phosphate availability [2–4]. In *Arabidopsis* under low Pi, the sites of iron (Fe) accumulation and callose deposition are determined by the LPR1-PDR2 modules in both the meristem and elongation zone of the primary root, via apoplastically located LPR1 activity. Callose deposition, which causes impaired movement of SHORT ROOT (SHR) and interferes with the symplastic communication, is responsible for root meristem differentiation [5]. Low Pi stress induces iron mobilization in RAM through the action of *LPR1/LPR2*, causing the expression of CLAVATA3/ENDOSPERM SURROUNDING REGION (CLE14) in the proximal meristem region. CLAVATA2 (CLV2) and PEP1 RECEPTOR 2 (PEPR2) receptors perceive CLE14 and trigger RAM differentiation in *Arabidopsis*, with concomitant down-regulation of both SHORT ROOT (SHR)/SCARECROW (SCR) and PIN/AUXIN pathways [6].

Recently, researchers increasingly focused on the mechanism underlying the rice response to low Pi. Pi deficiency causes a significant reduction in the net photosynthetic rate of rice plants [7]. Photosynthetic CO_2_ assimilation is decreased by Pi deficiency as a result of the decreased RuBP pool size in rice [8]. Pi deficiency affects diverse metabolic pathways most of which are related to glucose, pyruvate, sucrose, starch, and chlorophyll a in rice leaves [9]. The genes involved in Pi transport, phosphatases, and genes pertaining to both primary and secondary metabolism were affected differently by Pi deficiency in rice roots [10]. Phosphate over accumulator 2 (OsPHO2) knockout mutants indicates that OsPHO2, which functions downstream of the phosphate transporter traffic facilitator 1 (OsPHF1), modulates Pi utilization by regulating the expression of Pht1 transporters in rice [11]. The Phosphate Starvation Response Regulator 1 (PHR1) is a MYB transcription factor that plays a key role in Pi starvation signaling. OsPHR1 and OsPHR2 are homologous proteins of PHR1 in rice [12]. Overexpression of OsPHR2 in rice mimicked the Pi starvation signal. It induces Pi Starvation Induced (PSI), OsIPS1/2 (the gene encoding the signal molecules), miRNAs, SPX domain-containing protein (SPXs), phosphate transporter (PTs), and purple acid phosphatases (PAPs) gene expression, and results in enhanced Pi acquisition [12–17].

Root elongation induced by Pi deficiency has been reported as one of the adaptive mechanisms in plants. Enhanced external root efficiency or root growth may result in high phosphorus uptake from Pi-deficient soils. About 90% of Pi uptake was found as the result of enhanced root growth per unit root size in rice [18]. Studies have illustrated the inhibition of plant height, total dry weight, shoot dry weight, and root number under Pi deficiency, but the maximum yields of root length and root-shoot ratio were achieved by Pi-deficiency stress in rice [19]. A significant root elongation was indeed induced in rice under Pi-deficient conditions [20]. Root elongation clearly varied among different rice varieties screened under two different Pi levels [20, 21]. Genetic differences were found in rice root elongation under Pi deficiency, and a distinct quantitative trait locus (QTL) was reported on the long arm of chromosome 6 [22]. In addition, this QTL itself, or a tightly linked region, partly explains the decreased ability of excess iron accumulation in the shoots. The identified QTL would be useful in the improvement of rice varieties overcoming complex nutritional disorder caused by both Pi deficiency and iron-excess toxicity [20]. In the rice reference genome, as well as other phosphorus-starvation-intolerant modern varieties, phosphorus-starvation tolerance 1 (*PSTOL1*) was absent [23]. *PSTOL1* also played a role as an enhancer in early root-growth, thus enabling plants to acquire more phosphorus and other nutrients. In such varieties, overexpression of *PSTOL1* significantly enhanced grain production in phosphorus-deficient soil [24]. Overexpression of OsPHR2 led to Pi accumulation in rice leaves, as well as increases in root length, root-shoot ratio, and the number of root hairs [12]. Currently, OsWRKY74 is the unique confirmed WRKY gene which involved in the regulation of phosphate starvation response in rice. Transgenic seedlings overexpressing OsWRKY74 improved Pi uptake, length of roots, biomass, and iron accumulation levels, indicating that OsWRKY74 may be involved in the coordinate regulation of iron and Pi uptake [25].

Interestingly, Pi starvation induces the formation of reddish brown iron plaques on the surface of rice roots [26, 27], further promoting Fe accumulation in roots and shoots of rice plants [28]. However, the primary root and lateral root lengths both increase noticeably in tolerant rice cultivars under low Pi conditions [29]. This result suggests a different mechanism for the rice root morphological remodeling response to Pi deficiency compared to *Arabidopsis*. To date, the root morphological remodeling mechanism under low Pi in rice still remains unclear.

To illuminate whether Fe plays an important role in the regulation of rice root lengths under low Pi, the primary root length, Fe accumulation, and callose deposition in (or on) rice roots were investigated. Furthermore, Fe uptake, Fe distribution, and callose degradation-related gene expression were analyzed under low Pi conditions.

## Results

### Low pi led to root lengthening in rice

The effect of low Pi treatment (1/25 of a normal Pi supply level) on primary rice root length is shown in Table 1. Compared to the control (normal Pi supply level), the primary root length of rice cultivars Tongjing981 (TJ981) and ZhenDao 99 (ZD99) increased significantly (*P* < 0.05 and *P* < 0.01, respectively) after seedlings were treated in low Pi for 7, 15, and 30 d. However, primary root length change in ZhengHan 6 (ZH6) was either not significant (at 7 d) or significantly reduced (at 15 d); primary root length increased significantly when treated at low Pi for 30 d. These results indicate that low Pi stress promoted rice primary root lengthening, which is one of the main strategies of most rice cultivars to achieve acclimation to Pi deficiency. Apparently, the response pattern in root lengthening varied among different cultivars.

**Table 1:** Root length affected by low Pi treatment for 7, 15, 30 days

Samples		Treatment time(d)		
		7	15	30
TJ981	Normal P	8.40 ± 0.60	11.65 ± 0.37	24.77 ± 1.09
	Low P	9.73 ± 0.63**	13.98 ± 0.61**	42.15 ± 2.73**
ZH6	Normal p	6.83 ± 0.24	10.43 ± 0.46	22.23 ± 1.24
	Low P	7.09 ± 0.28	9.54 ± 0.56	31.93 ± 2.25**
ZD99	Normal P	7.14 ± 0.31	9.45 ± 0.47	25.46 ± 1.20
	Low P	7.67 ± 0.38**	11.18 ± 0.99**	39.38 ± 2.53**

“*” and “**” represent significant (*P* ≤ 0.05) and very significant difference (*P* ≤ 0.01) compared to control (the same applies hereinafter)

### Low pi promoted iron plaque formation on the rice root surface

**Fig. 1:**
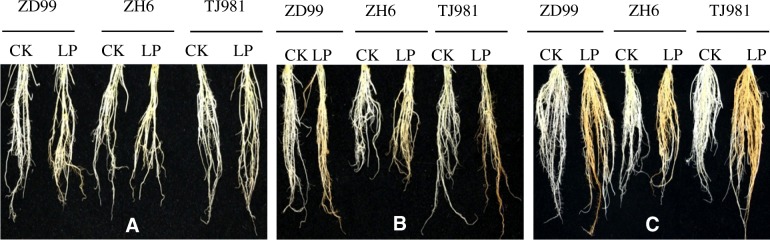
Formation of brown iron plaque on rice root surface in low Pi (LP) in comparison to control (CK). (**a**: low Pi treatment for 1d; **b**: low Pi treatment for 3 d; **c**: low Pi treatment for 15 d)

**Fig. 2:**
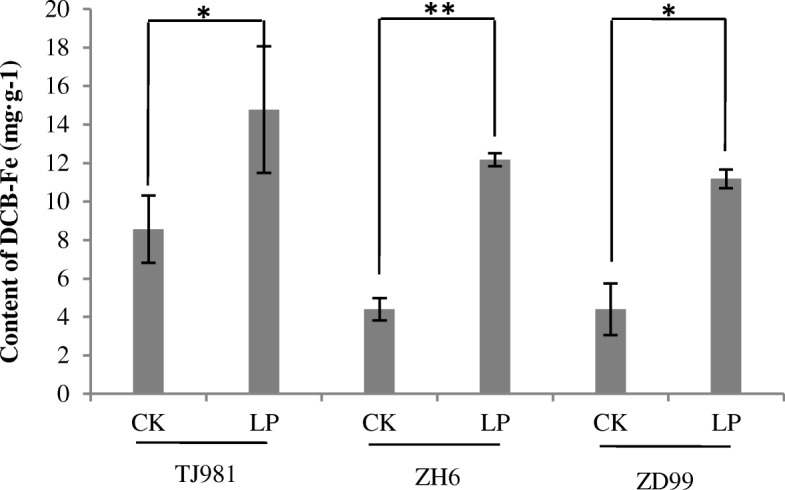
DCB-Fe content on root surface under low Pi treatment for 15 d. Notes: * indicates significant difference (*P* ≤ 0.05), ** indicates extremely significant difference (*P* ≤ 0.01)

DCB-Fe is the adsorption or precipitation of iron on the root surface. Consequently, a reddish-brown iron plaque on the rice root surface began to form after treatment by low Pi for 1d (Fig. 1a), and the thickness of iron plaque continuously increased with the prolonging of low Pi treatment time (Fig. 1b and c). The DCB-Fe contents increased either significantly or very significantly (Fig. 2) under the low Pi treatment for 15 d. Fe deposition on the rice root surface under low Pi treatment was confirmed by our results.

### Low pi induced *LPR1* genes expression

In *Arabidopsis*, the LPR1-PDR2 module mediates cell-specific Fe deposition in the cell walls of the RAM and elongation zone during Pi limitation. This provides evidence for apoplastic LPR1 ferroxidase activity and suggests that antagonistic interactions of Pi and Fe availability adjust the primary root growth rate via RAM-specific callose deposition, which is likely triggered by LPR1-dependent redox signaling [5]. In this experiment, the results of transcriptome sequencing showed that the expression of multicopper oxidase *LPR1 homolog 1–5* genes in the roots of three tested varieties was induced by low Pi treatment for 15 d (Table 2). Furthermore, the results of proteomic detection showed that the content of the LPR1 protein in low Pi treated rice roots was higher than that in the roots of control (data not shown). This suggests that the formation of Fe plaques on rice root surface was promoted by the induction of *LPR1* gene expression.

**Table 2:** The expression induction of LPR1 genes in rice roots treated by low Pi for 15 d

gene	Description	Log2FC		
		TJ981	ZD99	ZH6
*OS01G0126100*	Multicopper oxidase LPR1 homolog 1	1.274**	1.267**	1.036**
*OS01G0126200*	Multicopper oxidase LPR1 homolog 2	1.134*	0.550	1.305*
*OS01G0127000*	Multicopper oxidase LPR1 homolog 3	6.078**	2.453**	2.496*
*OS01G0126900*	Multicopper oxidase LPR1 homolog 4	2.055**	1.405**	0.275
*OS01G0127200*	Multicopper oxidase LPR1 homolog 5	2.180*	1.740**	1.153

Notes: * indicating the difference significant (*P* ≤ 0.05), ** indicating the difference extremely significant (*P* ≤ 0.01). The expression fold change (LP/ck) FC = 2^Log2FC^

### Low pi increased Fe content in the rice root symplast

**Fig. 3:**
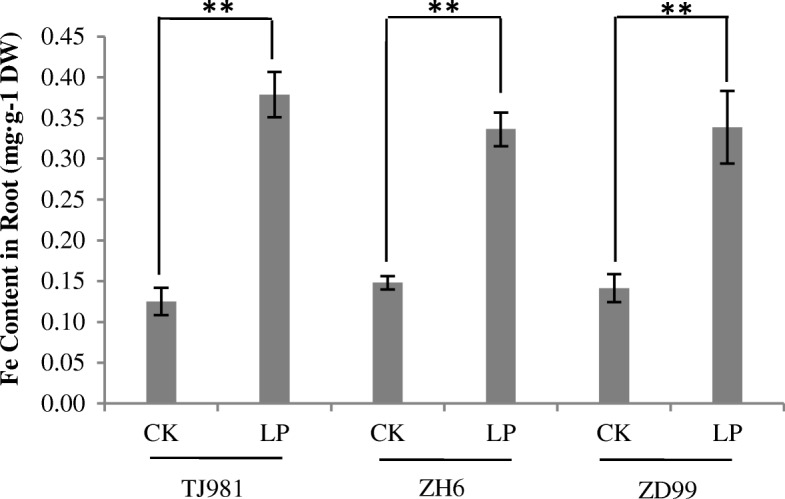
Effect of low Pi treatment on iron content in rice roots. Notes: ** indicates extremely significant difference (*P* ≤ 0.01)

Due to Fe deposition on the root surface, the Fe content increased very significantly in the root symplast of the three tested rice cultivars compared to the control (Fig. 3). It is interesting that the increased degree of Fe content in the root symplast was substantially lower than that deposited on the root surface. For example, in ZH6 cultivar Fe content on low Pi treated root surface increased by 7.77 mg compared to control (Fig. 2); however, it only increased by 0.19 mg in the ZH6 root symplast (Fig. 3). This result suggests that Fe uptake by the rice root symplasm might be limited under low Pi stress.

### Regulation of Fe accumulation in rice root symplasts under low pi stress

#### Gene expression regulation

##### Differential expression of Fe uptake related genes detected via transcriptome sequencing

The results basically clarified the existence of two distinct high affinity iron transport mechanisms in plants [30]. Non-graminaceous monocots and all dicots use the mechanism I Fe uptake strategy, while grasses use the mechanism II strategy. As a special case, rice may utilize both mechanism I and II Fe uptake strategies [31].

These experimental results indicate that the Fe uptake of mechanisms I and II was entirely inhibited by down regulating the expression of key enzyme encoding genes, associated with Fe uptake in rice roots under low Pi (Table 3).

**Table 3:** Effect of low Pi on transcriptional level of the iron absorption related genes detected via illumina expression profile sequencing

Ensemble_id	Description	TJ981		ZH6		ZD99	
		Log_2_FC	*P*-Value	Log_2_FC	*P*-Value	Log_2_FC	*P*-Value
*OS03G0667300*	Fe^2+^ transport protein 2 (IRT)	−2.683	5.00E-05	−3.766	5.00E-05	− 2.082	0.0003
*OS03G0307200*	Nicotianamine synthase 2 (NA2)	−2.731	5.00E-05	−5.639	5.00E-05	−2.353	5.00E-05
*OS03G0307300*	Nicotianamine synthase 1 (NA1)	−2.722	5.00E-05	−5.612	5.00E-05	−2.433	5.00E-05
*OS02G0306401*	Nicotianamine aminotransferase (NAAT)	−2.817	5.00E-05	−5.166	5.00E-05	−2.813	5.00E-05
*OS02G0650300*	Iron-phytosiderophore transporter (YSL15)	−2.474	5.00E-05	−4.732	5.00E-05	−2.695	5.00E-05
*OS02G0649900*	Metal-nicotianamine transporter (YSL2)	−4.446	5.00E-05	−4.201	5.00E-05	−2.774	5.00E-05
*OS04G0542200*	Probable metal-nicotianamine transporter (YSL9)	−1.485	5.00E-05	−1.419	5.00E-05	−0.694	–
*OS07G0258400*	Metal transporter Nramp1 (OsNRAMP1)	−2.134	5.00E-05	−3.658	5.00E-05	−2.479	5.00E-05
*OS05G0472400*	Zinc/iron permease family protein (ZIP)	−2.158	5.00E-05	−2.688	5.00E-05	−2.677	5.00E-05
*OS03G0811900*	ADP-ribosylation factor 1-like (ARF1)	−0.200	–	−1.043	5.00E-04	−0.706	–
*OS01G0265100*	ADP-ribosylation factor 2-like (ARF2)	−0.313	–	− 1.036	5.00E-04	−0.636	–
*OS01G0667600*	Ras-related protein RABA1f (RabGTPase)	−2.127	5.00E-05	−1.421	5.00E-04	−0.173	–
*OS08G0525000*	Ras-related protein RABA5c (RabGTPase)	−0.951	–	− 1.030	5.00E-05	−0.294.	–
*OS03G0843100*	ras-related protein RABA2a (RabGTPase)	−1.122	5.00E-05	−1.490	5.00E-05	−1.134	5.00E-05
OS09G0500900	Ferric reductase transmembrane domain containing protein (FR)	0.695	–	1.032	0.021	0.968	–
OS04G0538400	Vacuolar iron transporter2 (VIT2)	3.313	5.00E-05	1.504	5.00E-05	5.142	5.00E-05
OS09G0396900	Vacuolar iron transporter 1.2 (VIT1.2)	3.857	5.00E-05	2.403	5.00E-05	1.357	5.00E-05

Notes: The expression fold change (LP/ck) FC = 2^Log2FC^, e.g., the expression fold change (LP/ck) of *IRT* in TJ981 roots FC = 2^–2.683^ = 0.156. “-------” represents that due to FC ≤ 2 or ≥ 0.5, the *P*-Value was not given. *P*-Value ≤0.05 (or ≤ 0.01) represent that the difference reached significant (or very significant) levels, respectively

Although the expression of a ferric reductase transmembrane protein (FR) gene (*OS09G0500900*) in the ZH6 root was induced by low Pi, the expression of the Fe^2+^ transport protein 2 gene (*IRT, OS03G0667300*) was inhibited by low Pi in all three tested cultivars (Table 3), suggesting that low Pi reduced Fe^2+^ uptake by rice roots. Furthermore, the expression of nicotianaminesynthase *(NA2*, *OS03G0307200*; *NA1*, *OS03G0307300*) and nicotianamine aminotransferase A (*NAAT*, *OS02G0306401*) was down-regulated under low Pi (Table 3), showing that low Pi inhibited PS biosynthesis. Moreover, the expression of an ADP-ribosylation factor (such as *ARF1, OS03G0811900; ARF2, OS01G0265100*) and Rab GTPases (*such as RABA1f, OS01G0667600; RABA5c, OS08G0525000; RABA2a, OS03G0843100*) was down-regulated under low Pi (Table 3), suggesting that low Pi inhibited PS secretion. Furthermore, the expression of the Fe or metal-phytosiderophore transporter (*YSL15, OS02G0650300; YSL2, OS02G0649900; YSL9,OS04G0542200*) was all down-regulated due to low Pi (Table 3), indicating that low Pi also inhibited Fe^3+^-PS complex transportation.

Additionally, plants might also utilize a mechanism III iron absorption strategy. Moil (1999) reported that the metal transporter Nramp played an important role in the absorption of iron and other metal ions and suggested that plants may use a novel mechanism for phagocytic iron absorption. In this mechanism, Nramp can release Fe^2+^ from the endosome, then transferring it to the cytoplasm. Table 3 shows that low Pi down-regulated the expression of metal transporter Nramp1 (*NRAMP1, OS07G0258400*), indicating that the phagocytic mechanism of Fe^2+^ uptake is also inhibited by low Pi.

It is worth noting that the expression of the vacuolar iron transporter 2 gene (*VIT2, OS04G0538400*) and vacuolar iron transporter 1.2 (*VIT1.2, OS09G0396900*) was strongly induced by low Pi stress (Table 3), which suggests that the distribution of Fe in root cells was probably regulated by the expression of low-Pi-responsive genes.

#### The transcriptional level of differentially expressed genes verified via qRT-PCR

**Fig. 4:**
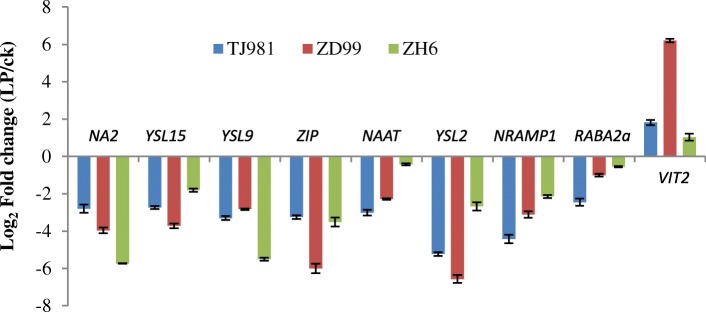
Transcriptional level of the differentially expressed genes verified via qRT-PCR. Expression fold change (LP/ck) FC = 2 Log2FC

To verify the transcriptome sequencing results, nine differentially expressed genes were selected and their transcriptional levels were tested via real-time fluorescent quantitative PCR (qRT-PCR) after rice seedlings were treated by low Pi for 15 d. The results (Fig. 4) show that the transcription of *NA2*, *NAAT*, *YSL15*, *YSL2*, *YSL9*, *NRAMP1*, *ZIP*, and *RABA2a* were down-regulated; however, the transcription of *VIT2* was up-regulated, which fully agrees with the results of transcriptome sequencing.

#### Effect of low pi and treatment time on the transcriptional level of key differentially expressed genes

Four key genes associated with Fe^3+^ uptake (*NA2*, *NAAT*, and *YSL15*) and intracellular distribution (*VIT2*) were selected to determine the effect of low Pi treatment time on the resulting transcriptional level. The results showed that the transcriptional levels of *NA2*, *NAAT*, and *YSL15* were inhibited by low Pi, and that inhibition of their transcription began after low Pi treatment for only 1 d. The transcriptionally inhibited degree of *NA2* increased with the prolonging of low Pi treatment time. However, the inhibited degree of *NAAT* and *YSL15* decreased slightly due to low Pi treatment for 5 d or 9 d; therefore, the first five days after low Pi treatment may form an emergency response stage; then, the inhibited degree increased again with low Pi treatment time further prolonging, which may be called an adaptive response stage. Nevertheless, low Pi induced the transcription of *VIT2* and the transcriptional induced degree of *VIT2* first increased, then slightly decreased with extended low Pi treatment time.

### Intracellular distribution regulation of Fe

**Fig. 5:**
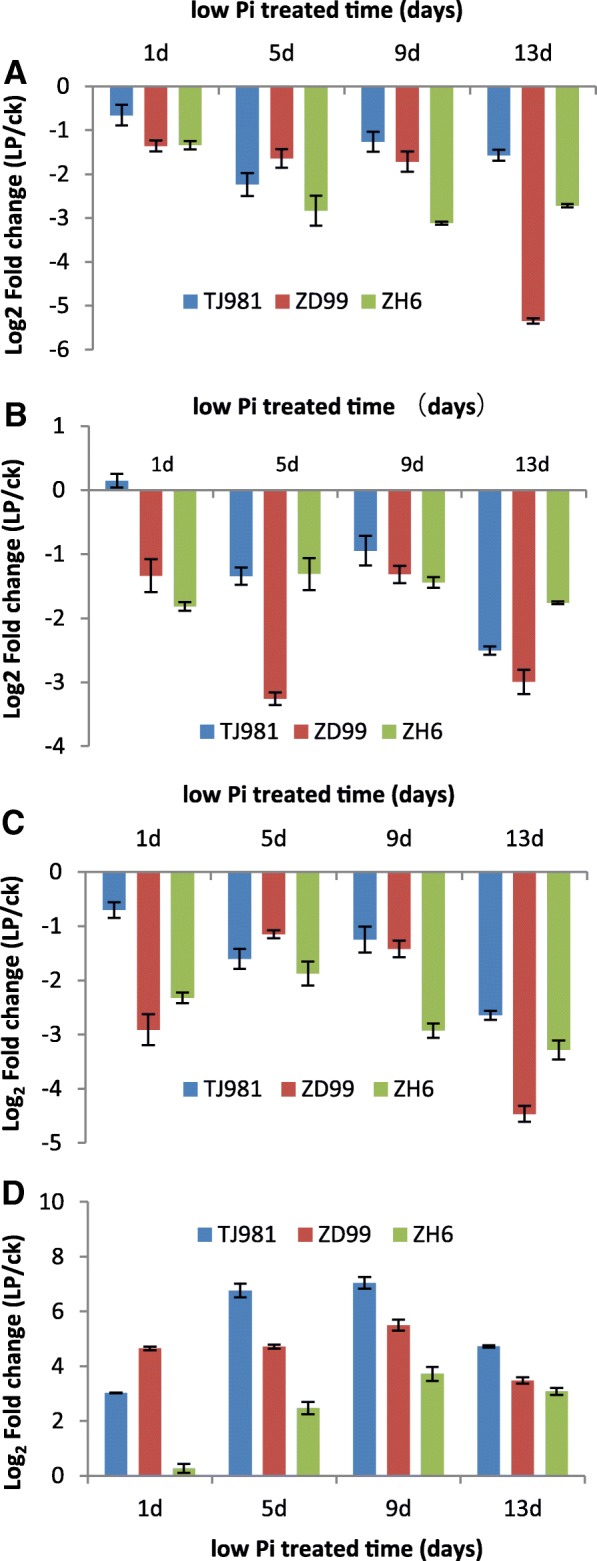
Effect of low Pi treatment time on the transcriptional level of Fe uptake-related Key genes. **a**: *OS03G0307200/NA2*, **b**: *OS02G0306401/NAAT*, **c**: *OS02G0650300/YSL15*, **d**: *OS04G0538400/VIT2.* Expression fold change (LP/ck) FC = 2 Log2FC

**Fig. 6:**
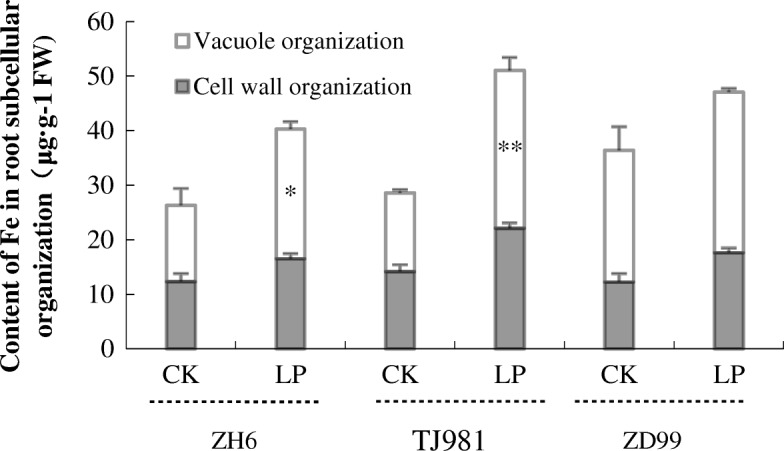
Effect of low Pi on the Fe content in subcellular organelles of rice root cells. Notes: * indicates significant difference (*P* ≤ 0.05), ** indicates extremely significant difference (*P* ≤ 0.01)

Although low Pi promoted Fe accumulation in rice roots (Fig. 2), the intracellular distribution of Fe still remained regulated. The Fe content in the vacuole of low Pi treated root cells was significantly higher than that in ck (Fig. 6), which was consistent with the expression induction of the VITs gene under low Pi (Table 3, Fig. 5d). Furthermore, the Fe content in the cell wall was also higher than that in ck (Fig. 6). These results suggest that Fe was mainly stored in root vacuoles and cell walls under low Pi treatment, to alleviate the toxic effect of excessive Fe in the cytoplasm.

In summary, Fe homeostasis in rice roots was regulated by coordinated Fe uptake, transport, and intracellular distribution under low Pi. In contrast to *Arabidopsis,* Fe accumulation in rice roots did not inhibit the primary root growth under low-Pi stress.

### Low-pi and low-Fe treatment leads to rice root lengthening

**Fig. 7:**
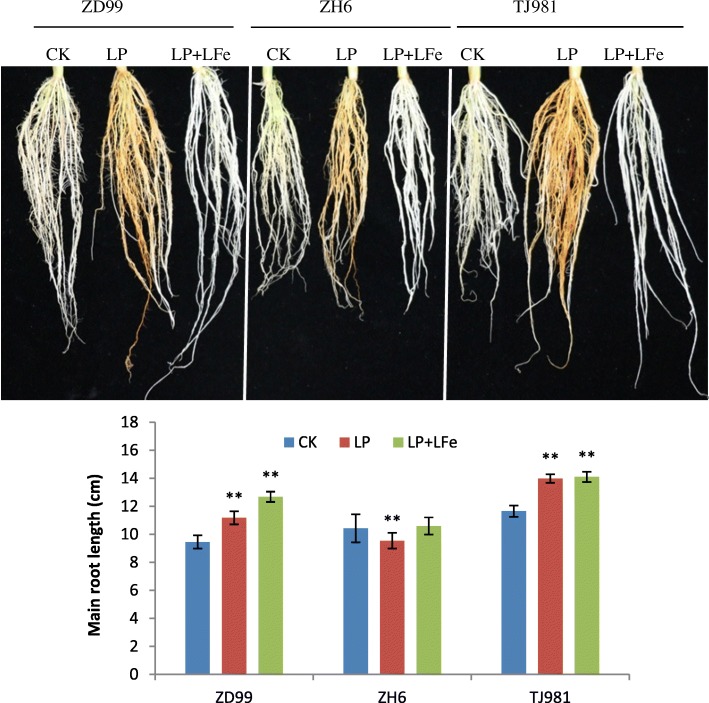
Effect of low-Pi and low-Fe on rice root length. Notes: ** indicates extremely significant difference (P ≤ 0.01)

As shown in Fig. 7, the low-Pi and low-Fe joint treatment (LP + LFe) did not cause the formation of Fe plaques on the rice root surface; however, the primary root lengths of TJ981 and ZD99 were significantly enhanced by either LP or LP + LFe treatments for 15 d compared to the control. This result indicates that the low Fe content in both medium and rice roots still resulted in the lengthening of the primary root, which was different in *Arabidopsis*.

### Low-pi promoted callose deposition in roots

**Fig. 8:**
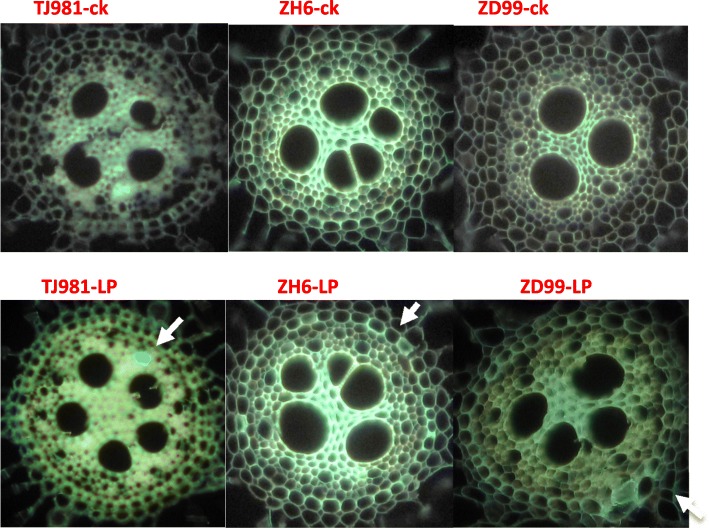
Effect of low-Pi on callose deposition in the elongation zones of primary roots. The arrows refer to the deposition of callose on the cell wall. The callose deposited on cell wall was dyed blue-green

In *Arabidopsis*, Pi limitation triggered cell-specific apoplastic Fe and callose depositions in both meristem and elongation zone of primary roots. Here, we showed that Low-Pi promoted a small callose deposition in the elongation zone of primary roots in rice (Fig. 8). However, the relative amount of callose deposition was smaller compared to *Arabidopsis*.

**Fig. 9:**
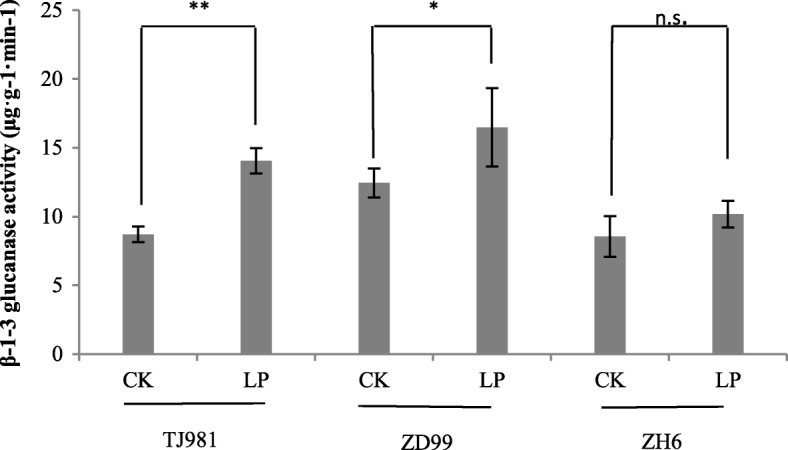
Effect of low-Pi on β-1-3 glucanase activity. “n.s.” and “*” represent non- significant and significant difference, respectively (*P* ≤ 0.05) compared to control. ** indicates extremely significant difference (*P* ≤ 0.01)

Callose hydrolysis is catalyzed by β-1-3 glucanase. The transcriptome sequencing results of this experiment showed that the expression of the β-1-3 glucanase gene was induced by low-Pi in TJ981 (*OS03G0221500*, Log_2_FC = 1.02, *P*-value = 5.00E-05) and ZH6 (*OS01G0631500*, Log_2_FC = 1.10, *P*-value = 0.00165) of roots. Furthermore, the β-1-3 glucanase activity in low-Pi treated rice roots was significantly higher than in control (Fig. 9). This result suggests that the callose deposition in low-Pi treated rice roots could be reversed by high expression of specific β-1,3 glucanase.

## Discussion

This study confirmed that Pi deficiency induced root morphological remodeling in rice, which is a major developmental plant response to Pi deficiency and has been suggested to enhance the plant’s adaptability to Pi deficiency. When cultured under Pi deficiency, some plants (such as *Arabidopsis*) decrease their primary root growth, while increasing the production of lateral roots [32]. However, unlike *Arabidopsis*, primary root lengthening happened during Pi deficiency treatment of rice [33, 34]. The results of this experiment confirmed that low Pi stress promoted rice root expansion (especially primary root lengthening).

Phosphorus deficiency induced reddish brown Fe plaque formation on the surface of rice roots [26, 27]. The Fe plaques that formed on the root surface of rice seedlings can be regarded as a nutrient pool, contributing to the uptake of P and Fe. Our results confirmed that the reddish-brown Fe plaques formed after low Pi treatment for 1 d (Fig. 1a), and the thickness of the Fe plaque continuously increased with prolonged low Pi treatment time (Fig. 1b and c). The formation of Fe plaques might be the result of the expression induction of *LPR1* genes.

When rice seedlings were treated with low Pi, the Fe content in root surface (apoplast) and root symplast increased significantly due to formation of the Fe plaque (Figs. 2 and 3). It has been reported that, in *Arabidopsis*, Pi limitation triggered apoplastic Fe and callose deposition in the root meristem, and callose deposition inhibited symplastic communication in the root stem cell niche, which subsequently inhibited primary root growth [5]. Therefore, the antagonistic interactions of Pi and Fe availability controlled the primary root growth of *Arabidopsis* via meristem-specific callose deposition. To date, the role of Fe in the rice root morphological remodeling response to low Pi remains unclear. Although low Pi increased the Fe contents both on root surface (apoplast) and in root symplast in rice, primary root lengthening was observed in this study, implying that rice used different regulatory mechanisms for root morphological remodeling under low Pi. Fe accumulation in rice roots did not inhibit primary root growth; in contrast, low Pi promoted primary root lengthening.

**Fig. 10:**
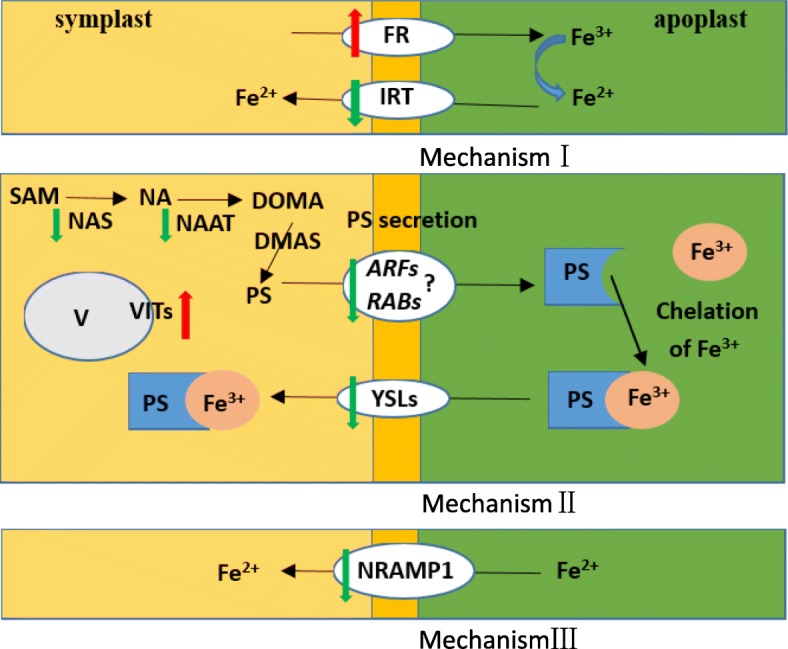
Fe uptake strategy for plants and effects of low Pi on the expression of key genes involved in Fe uptake and distribution in rice roots. The green arrows indicate the down-regulated expression of corresponding genes, while the red arrow indicates up-regulated expression of corresponding genes; V represents the vacuole

However, evidence for Fe-related toxicity during low Pi conditions is still missing. It has been proposed that the inhibited primary root growth under low Pi condition, might be caused by the toxic effect of excessive Fe [1]. Therefore, it is important to investigate how to regulate Fe homeostasis and alleviate the toxic effects of excessive accumulated Fe in low Pi treated rice roots. This experiment showed that, due to the down-regulated expression of Fe uptake-related key genes (including *IRT*, *NAS*, *NAAT*, *YSLs*, *NRAMP1*, *ZIP*, *ARFs*, and *RABs*) (Table 3, Fig. 10), the Fe uptake by mechanisms I, II, and III were all inhibited under low Pi stress. Furthermore, due to the up-regulated expression of the *VIT2* and *VIT1.2* genes (Fig. 10), Fe was stored more in the root vacuole and cell wall under low Pi stress. Therefore, Fe homeostasis in the rice root was appropriately controlled by Fe uptake, transport, and intracellular distribution. Consequently, Fe accumulation in the rice root symplast was insufficient to inhibit primary root growth under low-Pi stress. Moreover, LP + LFe treatment still induced primary root lengthening compared to control treatment. Consequently, Fe does not play an important role in rice root morphological remodeling under low Pi.

One of the toxic effects of Fe accumulation in low Pi treated rice roots was the triggering of callose deposition in the root meristem. Our experiment showed that a small amount of callose was deposited in the elongation zone of rice primary roots. However, the relative amount of callose deposition was small compared to that in *Arabidopsis*, which may consequently not be sufficient to interfere with intercellular communication. The reason for callose deposition under control conditions might be the expression induction and increased activity of β-1-3 glucanase in low Pi treated rice roots.

In summary, because Fe homeostasis in rice roots is appropriately controlled by the expression regulation of Fe uptake, transport, and intracellular distribution related genes, and because callose deposition in the cell wall is also controlled by expression induction and increased activity of β-1-3 glucanase, Fe only plays a small role in rice root morphological remodeling under low Pi. In contrast, low Pi promoted primary root lengthening.

## Conclusions

Pi deficiency induces root morphological remodeling in plants. This study confirmed that low Pi caused Fe plaque formation on the root surface and promoted primary root lengthening of rice. Fe uptake mechanisms I, II, and III in rice roots were all inhibited by down-regulated expression of Fe uptake-related key genes. Fe was increasingly stored in both root vacuoles and cell walls due to the up-regulated expression of the VITs gene and callose deposition in the cell wall was inhibited by induced expression and increased activity of β-1-3 glucanase. We also found that low Pi and low Fe treatment still caused primary root lengthening. All these results suggest that caused by the homeostasis of Fe and callose in rice roots treated to low Pi, Fe does not play an important role in rice root morphological remodeling under low Pi. In contrast, low Pi enhances primary root lengthening. However, the mechanism of low Pi promoting root length still remains unknown and it is significant to further elucidate the underlying mechanism.

## Methods

### Plant materials

Informed by our previous research results, three following rice cultivars were selected as test materials: TongJing 981 (TJ981), ZhengHan 6 (ZH6), and ZhenDao 99 (ZD99) corresponding to the primary root lengthening type, phosphorus efficient uptake and utilization type, and intermediate type rice cultivar response to low Pi, respectively.

### Rice seedling culture and treatment

Plump rice seeds were selected and sterilized via 10% H_2_O_2_ for 30 min. After washing with deionized water, the seeds were placed in a Petri dish (17 cm), filled with deionized water to accelerate germination at 32 °C. The germinating seeds were selected and placed into 96-well plastic plates. Then, the plates were placed in plastic boxes and complete nutrient solution of the International Rice Research Institute [containing: 1.45 mM NH_4_NO_3_,0.323 mM NaH_2_PO_4_•2H_2_O, 0.512 mM K_2_SO_4_, 0.998 mM CaCl_2_, 1.643 mM MgSO_4_ •7H_2_O, 9.1μΜ MnCl_2_•4H_2_O, 0.075 μM (NH_4_)_6_Mo_7_O_24_•4H_2_O, 18.882 μM H_3_BO_3_, 0.152 μM ZnSO_4_ •7H_2_O, 0.155 μM CuSO_4_•5H_2_O, 0.036 mM FeCl_3_•6H_2_O, and 0.031 mM Na_2_EDTA•2H_2_O, 0.071 mM Citric acid monohydrate, and 500 ml of concentrated sulfuric acid were added every 10 L; (pH = 5.4)] was added. When the seedlings had grown to the 3-leaf stage, healthy seedlings were chosen and cultured with either normal nutrient solution (CK), low Pi (LP), or low Fe (LFe) nutrient solution. The Pi concentration of LP/CK was 1/25, while the Fe concentration of LFe/CK was 1/20. Each treatment contained six biological replicates. The seedlings were further cultured in an artificial climate chamber under controlled conditions (14-h photoperiod, 75% relative humidity, and 32/27 °C day/night regime). The solution was changed daily and the pH was adjusted to about 5.1. Rice seedlings were sampled after treatment durations of 1 to 30 days.

### Extraction of DCB-Fe from the rice root surface

DCB-Fe is a general term for both adsorption and precipitation of Fe on the root surface. DCB-Fe was measured via DCB (dithionite-citrate-bicarbonate) extraction method [35]. Briefly, after low Pi treatment for 15 d, the rice roots were sampled and soaked overnight using tap water. After repeated washing with deionized water, the root surface moisture was absorbed by absorbent paper and the roots were placed in 150 ml triangular flasks. The DCB extraction solution (consisting of: 40 ml of 0.3 mol/L Na_3_C_6_H_5_O_7_·2H_2_O, 5.0 ml of 1.0 mol/L NaHCO_3_, and 3.0 g Na_2_S_2_O_4_) was added to triangular flasks, and then oscillated on a 280x g shaking table for 3 h at 25 °C. The solution was filtered into 100 ml volumetric flasks at constant volumes. DCB-Fe content (or iron plaque thickness) was verified via the iron content of the per unit dry weight of roots.

### Digestion of rice roots

After iron plaque removal via the DCB extraction method, the roots were repeatedly rinsed with deionized water, dried in the oven at 70–80 °C, and ground to a fine powder in a ceramic mortar. Then, 0.5 g root powder was weighed, and both 5 ml concentrated nitric acid and 3 ml deionized water were added. After H_2_O_2_ addition (two drops), the root powder was digested in a high-pressure closed microwave digestion instrument (MARS 6, CEM, USA). The digestion solution was transferred to a 50 ml volumetric flask at constant volume.

### Subcellular structure separation

After iron plaque removal, 1.0 g roots were weighed and placed in a pre-cooling mortar. 10 mL homogenate (consisting of: 0.25 mol/L sucrose, 50 mmol/L Tris-maleate buffer (pH = 7.8), 1 mmol/L MgCl_2_ and 10 mmol/L cysteine) were added to the mortar. The roots were ground to a fine homogenate, which was then transferred into a 50 mL centrifuge tube, and centrifuged using a high-speed refrigerated centrifuge at 1000 x g for 2 min at 4 °C. The precipitation at the bottom was collected for the cell wall component. The supernatant was further centrifuged at 12000 x g for 30 min at 4 °C. The fragments on the bottom were collected for evaluation of the organelle composition. The supernatant formed the vacuolar component (consisting of vacuole and cytoplasm Fe).

### Determination of iron content

The contents of DCB-Fe, Fe in roots, and subcellular Fe (consisting of cell wall, organelle, and vacuolar components) were determined via plasma-atomic emission spectrometry (iCAP-6300, Thermo Fisher SCIENTIFIC, USA).

### Observation of callose deposition

To measure callose deposition, the method of frozen section with aniline blue fluorescent staining was used [36]. Briefly, 10 mm rice root tips were sampled and a 5 mm subparagraph was cut out. The root tips were immersed in 10% glycerin. After pumping gas for 15 min, the root tips were embedded, fixed, and cut into 15 μm slices using a Leica CM 1900 frozen microtome. The sections were placed on a slide and soaked in 95% ethanol solution overnight; then, soaked in phosphate buffer (l/15 mol/L, pH = 7.0) for 30 min. The sections were dyed in 0.05% aniline blue for 60 min. The deposited callose was observed with an OlympusBX51 fluorescence microscope, excited by ultraviolet light.

### Determination of β-1-3 glucanase activity

Determination of β-1-3 glucanase activity was conducted in accordance with Zhang et al. [37]; however, a slight change was implemented: 0.5 g roots were weighed and placed in a pre-cooled mortar. 5.5 mL sodium acetate buffer (0.05 mol/L, pH = 5.0) were added to the mortar. The roots were ground to a homogenate, which was then transferred into a 10 mL centrifuge tube and centrifuged at 15000 r/min for 15 min at 4 °C. The supernatant was used as enzyme extraction. The enzyme extraction was heated in a water bath at 100 °C for 10 min, which was used as control. 100 μl Okam solution (1 mg/mL) and 100 μl enzyme extraction was added to a 5 ml centrifuge tube, and heated in a water bath at 37 °C for 30 min. Then, 1 ml DNS solution was added to terminate the reaction. The reaction solution was placed in a boiling water bath for 5 min of coloration. After cooling to room temperature, the amount of glucose was measured via colorimetry at 540 nm.

### Transcriptome sequencing

#### RNA library construction and sequencing

For mRNA library construction and deep sequencing, RNA samples were prepared via the TruSeq RNA Sample Preparation Kit according to the manufacture’s protocol [38]. Briefly, the poly-A containing mRNA molecules were purified with 3 μg of total RNA via poly-T oligo-attached magnetic beads. Cleaved RNA fragments were reversely transcribed into first strand cDNA using random hexamers, followed by second-strand cDNA synthesis using DNA polymerase I and RNase H. cDNA fragments were purified, end blunted, ‘A’ tailed, and adaptor ligated. PCR was used to selectively enrich DNA fragments with adapter molecules on both ends and to amplify the amount of DNA in the library. The number of PCR cycles was minimized to avoid skewing representation of the library [39]. The resulting library was qualified via the Agilent 2100 bioanalyzer and quantified via both Qubit and qPCR. The produced libraries were sequenced on the HiSeq 2500 platform.

#### Data analysis workflow of transcriptional profiling

Information on the reference gene set and corresponding annotations: *Oryza indica* gene set referred to ENSEMBL (ftp://ftp.ensemblgenomes.org/pub/-release-23/plants/fasta/oryza_indica/cdna/Oryza_indica.ASM465v1.23.cdna.all.fa.gz).

Analysis of the gene expression profile: sequencing reads were mapped onto the reference gene set via Bowtie1 software (Bowtie parameter: –v 3 –all –best –strata). A Perl script program was utilized to process the mapping results and to generate a gene expression profile.

#### Analysis of differentially expressed genes

According to credibility interval approaches that had been reported for the analysis of SAGE data5 [40], the edgeR6 program was used to identify differentially expressed mRNAs based on their relative quantities, which were reflected by individual gene reads [41]. The method used empirical Bayes estimation and exact tests based on negative binomial distribution. Genes with a *P* value ≤0.01 and an expression ratio ≥ 2 (up-regulation) or expression ratio ≤ 0.5 (down-regulation) were recognized as significantly differentially expressed genes between both samples.

### Real time fluorescent quantitative PCR (qRT-PCR) verification

#### Primer design and synthesis

Nine differentially expressed Fe uptake and distribution-related genes detected via RNA-seq were selected. cDNA sequences of these genes were searched in a NCBI database. Primers (see Table 4) were designed with Primer 5.0 software according to CDS and then synthesized by Invitrogen Co. Ltd., USA.

**Table 4:** Primers for real-time quantitative PCR

Gene Symbol	Sense primer (5′-3′)	Reverse primer (5′-3′)	Product length	Tm
*OS07G0258400*	TTTGGGTGATTTTGATTGGC	CTTCTGGAATATCGGAAGCA	180	55.00 54.94
*OS05G0472400*	TTTCTTGCTCTAAGCAGTGT	CCACAAAAAGTCTACACCCA	169	54.91 55.49
*OS04G0542200*	CAAGACGGGACATCTAACAT	AGGCACTGTAGAACAAGAAG	116	54.87 55.01
*OS03G0843100*	ATTGGATTGCTTGAGGTGAT	GAAGCGGCTGTACTATGTTA	130	54.97 55.00
*OS03G0307200*	TGAGTGCGTGCATAGTAATC	TCATCCACACAACAAGAACA	122	55.67 55.12
*OS02G0306401*	GTTTGCCTTTTATGGCCTTT	CACTATATATGGCTCGCCTC	105	54.99 55.01
*OS02G0650300*	GAAAGCAGCATGACAAGTTT	AAAAACGACTGCAAAAGGAG	127	55.08 55.02
*OS02G0649900*	TCCTTAACTTGCTTCCACTC	GGAAGAAGCTCCATAAGAGG	183	54.99 54.93
*OS04G0538400*	AATAATCAAGGGGTTGTGCT	AACCATTACACTTACACCCC	142	54.88 54.94

#### Total RNA isolation

After the rice seedlings had been treated by low Pi for 1, 5, 9, 13 days, roots were harvested to extract total RNA using the RNAprep pureplant kit (Tiangen, Beijing, China), according to the manufacturer’s protocol.

#### First-strand cDNA synthesis

First-strand cDNA was synthesized by reverse transcribing 5 μL of total RNA in a final reaction volume of 20 μL using TIANScriptRT kit (Tiangen, Beijing, China) according to the manufacturer’s instructions. The cDNA concentration was determined using an Eppendorf Biophotometer. According to the cDNA concentration, the volumes of the products of reverse transcription were regulated to ensure identical cDNA concentration in each treatment.

#### Real-time quantitative PCR detection

Real-time quantitative PCR analysis was conducted using the Real-Time PCR System (CFX96 Touch, Bio-Rad, USA). The SYBR Premix Ex Taq (TaKaRa) kit was used, using ubiquitin 5 (UBQ 5) gene as reference gene [42]. Amplification was done in parallel with the target gene allowing normalization of gene expression, while providing quantification. The reaction procedure was as follows: Pre-denaturation at 95 °C for 30 s, followed by 40 cycles of: denaturation at 95 °C for 5 s, annealing at 55 °C for 30 s, and extension at 70 °C for 30 s. The relative expressed quantitation (RQ) was calculated via the 2^−ΔΔCT^ method [43].

### Data statistical analysis

All data were analyzed with Excel 2003 and SPSS 12.0 using AVOV at a significance level of *P* ≤ 0.05.

## Abbreviations

ARF: ADP-ribosylation factor
CK: Normal nutrient solution
DCB: Dithionite-citrate-bicarbonate
DMAS: Deoxymugineicacid synthase
DNS: 3,5-dinitrosalicylic acid colorimetry
DOMA: Deoxymugineic acid
FR: Ferric reductase transmembrane protein
FRO: Ferric reductase oxidase
IRT: The Fe^2+^ transporter
LFe: Low Fe
LP: Low Pi
NA: Nicotianamine
NAAT: Nicotianamine aminotransferase
NAS: Nicotianamine synthases
Pi: Phosphorus
PS: Phytosiderophores
qRT-PCR: Quantitative Real-time Polymerase Chain Reaction
RAM: Root apical meristem
SAM: S-adenosylmethionine
TJ981: TongJing 981
VIT2: Vacuolar iron transporter2 gene
ZD99: ZhenDao 99
ZH6: ZhengHan 6
